# Movements and Habitat Use of an Endangered Snake, *Hoplocephalus bungaroides* (Elapidae): Implications for Conservation

**DOI:** 10.1371/journal.pone.0061711

**Published:** 2013-04-22

**Authors:** Benjamin M. Croak, Mathew S. Crowther, Jonathan K. Webb, Richard Shine

**Affiliations:** 1 School of Biological Sciences A08, University of Sydney, Camperdown, New South Wales, Australia; 2 School of the Environment, University of Technology Sydney, Broadway, New South Wales, Australia; State Natural History Museum, Germany

## Abstract

A detailed understanding of how extensively animals move through the landscape, and the habitat features upon which they rely, can identify conservation priorities and thus inform management planning. For many endangered species, information on habitat use either is sparse, or is based upon studies from a small part of the species’ range. The broad-headed snake (*Hoplocephalus bungaroides*) is restricted to a specialized habitat (sandstone outcrops and nearby forests) within a small geographic range in south-eastern Australia. Previous research on this endangered taxon was done at a single site in the extreme south of the species’ geographic range. We captured and radio-tracked 9 adult broad-headed snakes at sites in the northern part of the species’ distribution, to evaluate the generality of results from prior studies, and to identify critical habitat components for this northern population. Snakes spent most of winter beneath sun-warmed rocks then shifted to tree hollows in summer. Thermal regimes within retreat-sites support the hypothesis that this shift is thermally driven. Intervals between successive displacements were longer than in the southern snakes but dispersal distances per move and home ranges were similar. Our snakes showed non-random preferences both in terms of macrohabitat (e.g., avoidance of some vegetation types) and microhabitat (e.g., frequent use of hollow-bearing trees). Despite many consistencies, the ecology of this species differs enough between southern and northern extremes of its range that managers need to incorporate information on local features to most effectively conserve this threatened reptile.

## Introduction

Human-induced fragmentation of landscapes and habitats can lead to a reduction in biodiversity [1], [2]. Although species that are able to exploit a variety of habitats may be relatively insensitive to habitat disturbance [3], species that have evolved behaviors or physical traits that facilitate reliance on specialized habitat use may find altered habitats difficult or impossible to occupy [3]–[5]. For such species, disturbance of critical habitat can lead to endangerment or extinction [3]. Life history traits also influence a species’ ability to tolerate degradation of preferred habitat type; for example, taxa with small population sizes and low rates of reproduction and dispersal may be at particular risk [6], [7].

To conserve highly specialized animals, we need detailed information on habitat use, dispersal and movement patterns [8]. Unfortunately, such data often are laborious to collect, especially for endangered species – both because they are rare, and because research methods must not inflict additional stress [9]. As a result, our knowledge on many endangered taxa is based on studies that have been performed at only a single site (where researchers can most easily obtain and study animals: [10], [11]). Often, such sites are atypical of conditions that pertain over most of the species’ range [10], [11]. Indeed, a disproportionate reliance on studies on a small and unrepresentative series of populations is a general problem in ecological research: much of what we know about even widely-distributed lineages is based upon multiple studies on a small number of populations (e.g., gartersnakes in Manitoba: [12]). This is especially worrying for endangered-species research, because logistics may make studies elsewhere almost impossible.

One such species is the broad-headed snake (*Hoplocephalus bungaroides*), an elapid species that has drastically declined since European settlement of Australia [13]–[16]. Broad-headed snakes rely on specific habitat attributes; they shelter beneath thin, sun-exposed exfoliated rocks on sandstone rock outcrops with western or north-western aspects [17]. These retreat sites allow snakes to thermoregulate during winter and spring. *Hoplocephalus bungaroides* also exhibit other life history traits that render them vulnerable to disturbance e.g., dependence on high rates of adult survival, infrequent breeding (every 3 to 4 years), low fecundity (3 to 4 offspring per litter), late maturity (up to 6 years), low rates of dispersal and a small geographic range. All of these traits contribute to the endangered status of *H. bungaroides* [18]. Also, the habitat of *H. bungaroides* has become fragmented, and subject to vegetation overgrowth [19]–[21] and removal of shelter-sites (exfoliated rock) for landscaping and gardening [15], [22]–[24].

To date, most research on *H. bungaroides* has been conducted on a single population in the extreme south of the species’ range [17], [18], [23]–[25]. Genetic data show that this intensively-studied population belongs to a genetically distinct clade, with another isolated, evolutionarily significant unit identified in the north of the species range. Those two clades diverged approximately 800 000 years ago [26]. Vegetation, temperatures and potential prey species differ between the northern and southern parts of the species’ range [27]. In the current paper, we describe habitat use and movements of snakes from the previously unstudied northern clade.

## Materials and Methods

### Ethics Statement

The University of Sydney Animal Care and Ethics Committee specifically approved this study and provided permits specifically for this project (L04/12-2008/3/4927). All work with live animals followed the approved ethical protocols. Snakes were collected by hand, and returned to the laboratory in clean cloth bags (individually) in insulated containers. They were maintained in individual enclosures with access to heating, shelter and food (see below). All surgical procedures were performed by trained veterinary surgeons, and snakes were carefully monitored post-operatively prior to release into the field at their original capture site. Prior to surgery snakes were administered morphine to relieve pain. No snakes were killed during the study, and all were alert and active when released at the conclusion of the work.

### Study Species

*Hoplocephalus bungaroides* are medium sized (to 90 cm: [27]), brightly colored ambush predators [28]. During winter the snakes live in thermally suitable crevices that form between thin, exfoliated rock and parent bedrock that is exposed to afternoon sun [17]. During the warmer parts of the year, these exposed rock exfoliations become too hot and snakes move into tree hollows in adjacent woodlands [21]. This habitat specificity means that *H. bungaroides* are restricted to areas that provide access both to sun exposed rock-on-rock exfoliations, and to suitable areas of surrounding forest [17], [21].

### Study Sites

Yengo and Wollemi National Parks are 100 km north-west of Sydney. We radio-tracked snakes at one study site inside Wollemi National Park (NP), and at two study sites inside Yengo NP. All sites were approximately 2 km apart, and consisted of exposed Hawkesbury sandstone outcrops surrounded by open eucalypt woodland dominated by Sydney peppermint (*Eucalyptus urceolaris*), narrow-leafed stringy-bark (*Eucalyptus sparsifolia*), yellow bloodwood (*Corymbia eximia*), red bloodwood (*Corymbia gummifera*), grey gum (*Eucalyptus punctata*), and scribbly gum (*Eucalyptus haemastoma*).

### Capturing Snakes

To track *H. bungaroides* in the spring/summer period of 2010/2011 and 2011/2012 we captured nine snakes during late winter of 2010 and 2011. Snakes are accessible at this time of year because they shelter beneath thin rock exfoliations that are easily lifted and replaced. We captured seven snakes in Wollemi NP and two snakes in Yengo NP. We tracked two of the snakes caught in Wollemi NP and the two snakes caught in Yengo NP over the spring/summer of 2010/2011 (20/10/2010 to 07/02/2011). We tracked one of the snakes from Yengo NP, one of the snakes from Wollemi NP and an additional snake captured in Wollemi NP during the winter of 2011 from 10/05/2011 to 11/08/2011. We tracked the remaining four snakes in Wollemi NP over the spring/summer of 2011/2012 (16/11/2011 to 16/01/2012: see Table 1). We captured all snakes by hand and placed them in cotton bags for transportation to the laboratory. We housed snakes individually in plastic containers (31×22 cm, 10 cm high, containing a shelter and water dish) in a 12∶12 light:dark regime and constant temperature of 19°C. We placed a heat mat under one end of the enclosure to allow snakes to thermoregulate. We fed the snakes fortnightly on frozen-then-thawed laboratory mice. We transported snakes to an approved veterinarian as per animal ethics protocol L04/12-2008/3/4927 for surgical implantation of transmitters (BD-2T, Holohil Systems, Carp, Ontario, Canada). We recaptured snakes prior to signal failure so that we could surgically remove the transmitters.

**Table 1:** Home ranges of radio-tracked broad-headed snakes, *Hoplocephalus bungaroides*, at sites in the extreme north of the species’ range.

ID	Site	Sex	SVL (mm)	Mass (g)	Season	Home Range (ha)
Snake 1	Y	M	550	50.5	S10/11	9.43
Snake 3	Y	F	565	52.0	S10/11	6.36
Snake 4	W	F	565	51.5	S10/11	9.89
Snake 5	W	F	670	62.0	S10/11	1.39
Snake 3	W	F	565	52.0	W 11	0.09
Snake 5	W	F	670	62.0	W 11	0.83
Snake 7	W	M	555	51.0	W 11	0.57
Snake 6	W	F	672	63.5	S11/12	1.22
Snake 9	W	F	554	50.0	S11/12	0.01
Snake 11	W	M	570	52.0	S11/12	2.43
Snake 12	W	M	650	62.0	S11/12	0.24

“Season” shows season and year: for example, “S10/11” = spring and summer of 2010–2011. SVL = snout to vent length; M = male; F = female; W = Wollemi National Park; Y = Yengo National Park; S10/11 = spring/summer tracking period of 2010–2011; S11/12 = spring/summer tracking period of 2011–2012; W11 = winter tracking period of 2011. Home ranges were estimated using the minimum convex polygon method in ARC GIS 9.3.

### Surgical Methods

All surgeries were carried out by a qualified veterinarian. Each snake was examined and weighed, then pre-medicated with morphine 1 mg. kg^−1^ intramuscularly 10 min prior to induction. Snakes were induced with alfaxan 10 mg. kg^−1^ (intramuscular, or injected into the tail vein). Once the snake was anesthetized, a mask made from a 10 ml syringe was placed over the snake’s head (held in place with transpore tape) to provide a mixture of isoflourane and oxygen for anesthesia. Transmitters were cold-sterilized in a solution of F10 and water, and scales/skin were prepared using chlorhexidine scrub followed by an iodine spray. The transmitter aerial was trimmed to fit within the snake’s body.

A scalpel was used to make a small incision 20 mm above the vent, and then alligator forceps were used to blunt-dissect against the body wall up to a point two-thirds of the way up the snake’s body. A second incision was made over the tip of the alligator forceps and the transmitter antenna was grasped with the forceps and pulled through the coelom such that the aerial sat flat within the body cavity. The transmitter body was then introduced into the coelom. Both incisions were closed with 3-0 premilene non-absorbable suture material. A mixture of 41% warm water, 9% saline and 50% Hartmann’s fluids were then injected subcutaneously at a dose of 3% body mass. To remove transmitters, the above procedure was reversed. No adverse effects were noted from surgery, and we released all snakes within one week after surgery.

### Tracking Snakes

We tracked snakes twice per week during spring and summer in 2010–2011 (October to February) and 2011–2012 (November to February). We tracked snakes once per week during winter 2011. We used a hand-held UHF tracking receiver (Australis 26K, Titley Scientific, QLD, Australia) fitted with a Yagi antenna, and recorded location data using a hand-held global positioning system (GPS) device (GPSMAP 76, Garmin International, Olathe, KS, USA). We quantified attributes of trees used by snakes as retreat sites in spring/summer, plus five randomly chosen nearby trees (see analysis of microhabitat use by snakes). We also quantified the thermal regime of rocks used by snakes in winter (see seasonal shifts in thermal regimes within retreat sites).

### Analyses of Snake Movements

We used ARC GIS 9.3 (Esri, Redlands, CA, USA) to calculate the total distances moved by snakes throughout the study (m), the mean distance per move (displacement <1 m) and the time interval between moves (moves. day^−1^). We also calculated moves per tracking day; that is, the number of displacements divided by the number of radio-tracking days. We used a two-factor analysis of variance (2-way ANOVA) to test the effect of year and sex on these variables.

### Analyses of Snake Home Ranges

We imported GPS points of snake retreat sites into ARC GIS 9.3 and estimated home range sizes using the minimum convex polygon method [29], [30], to allow comparison with previous studies [17]. We imported layers (on vegetation types, elevation, waterways and roads and access points) to facilitate visual interpretation of habitat types.

### Analyses of Macrohabitat Use by Snakes

Using Student’s two sample *t*-tests, we compared tree characteristics (number of hollows, tree diameter at breast height [DBH; mm]) and tree height (m) in the vegetation structure types most often used by snakes (“Hawkesbury–Hornsby plateau exposed woodland” and “Mellong sandmass dry woodland”; see home ranges in Results section 3.2) to those in a widespread adjacent but non-used vegetation type (“Hawkesbury sheltered dry forest”; see home ranges in results section).

### Analyses of Microhabitat Use by Snakes

We compared the following characteristics of trees used by snakes to those of five nearby (unused) trees: species, alive or dead, DBH (mm), tree height (m), the number of visible hollows large enough to accommodate snakes, and height above-ground of the lowest hollow (m).

We used a generalized mixed effects model (GLMM: [31]) in the R package ‘nlme’ [32] with a binomial distribution to compare the characteristics of used trees to nearby trees, with individual snake being the random variable. GLMMs account for the non-independence of multiple measurements from each snake in resource selection models [33]. To assess which models best fitted the data, we ranked models, using all combinations of the variables, by the AICc. Any model with a ΔAICc <4 was considered a good fit to the data [34].

Some arboreal retreat-sites were inaccessible to us, but where feasible we measured thermal regimes inside used and unused hollows by attaching thermal data loggers (iButtons, Maxim Integrated, Sunnyvale, CA, USA) to lengths of wire, and inserting these as far as possible (typically, 10–50 cm) into hollows. We compared thermal data collected from used versus unused hollows with a repeated-measures ANOVA, with time of day as the repeated measure, and year and “used or not” as factors. We also compared maximum temperatures experienced within hollows of used versus unused trees using a Student’s paired *t*-test.

### Seasonal Shifts in Thermal Regimes within Retreat-Sites

Our radio-tracked snakes consistently used crevices beneath sun-warned rocks as winter retreat-sites, and hollows within trees as spring/summer retreat-sites. Thus, we compared thermal regimes under rocks with those in tree hollows (in both summer and winter), to compare the conditions that are available to snakes inside these types of shelter-sites at different times of year. We used thermal data loggers to measure temperatures under five rocks and four tree hollows (see above) used by *H. bungaroides* throughout 2010, 2011 and early 2012. We used one-way ANOVA to compare the total number of hours over both summers that 17 tree hollows and 16 rocks exceeded 32°C, the VTMax (Voluntary Maximum Temperature) for *H. bungaroides* [18]. The CTMax (Critical Maximum Temperature) has not been determined for *H. bungaroides*, so our analyses of this parameter were based on an estimate for the closely-related tiger snake, *Notechis scutatus* (38.0°C: [35], [36]).

### Spatial Ecology in the North *versus* the South of the Species’ Range

We compared home range size, mean distance per move and total distance moved of snakes tracked in the north to those of snakes tracked in the south during a study conducted over the spring/summer of 1992–93, 1993–94 and 1994–95 [17].

## Results

### Analyses of Snake Movements

**Figure 1:**
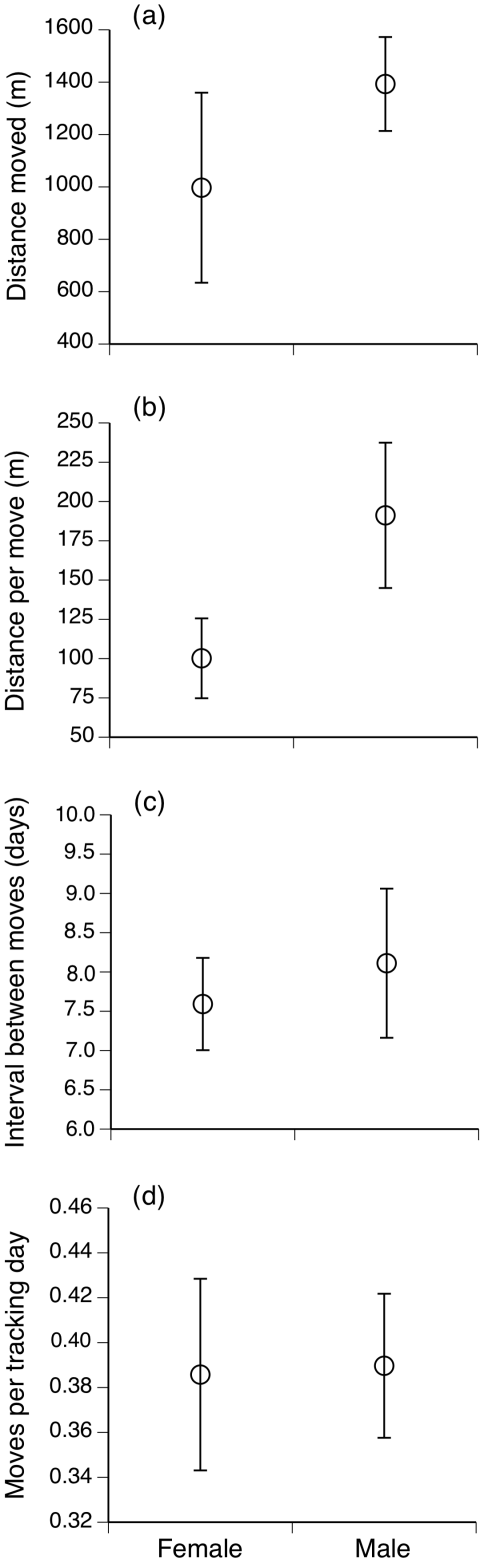
Movement data for broad-headed snakes collected over the Australian spring and summer seasons of 2010/2011 and 2011/2012. (a) mean total distance (in meters) moved by male and female snakes, (b) mean distance moved in meters per location shift by male and female snakes, (c) mean number of days between location shifts by male and female snakes and (d) the number of moves per tracking day for male and female snakes. All graphs show mean values and associated standard errors.

We found no significant effect of sex or year on total distances moved by our radio-tracked snakes (sex: *F*_1,5_ = 1.64, *P* = 0.26; year: *F*_1,5_ = 2.71, *P* = 0.16; Fig. 1a), nor on mean distance per move (sex: *F*_1,5_ = 3.59, *P* = 0.12; year: *F*_1,5_ = 0.37, *P* = 0.57; Fig. 1b), interval between successive moves (sex: *F*_1,5_ = 0.24, *P* = 0.65; year: *F*_1,5_ = 0.10, *P* = 0.77; Fig. 1c) or moves per tracking day (sex: *F*_1,5_ = 0.05, *P* = 0.83; year: *F*_1,5_ = 0.44, *P* = 0.54; Fig. 1d). No interactions between year and sex were statistically significant (i.e., all *P*>0.05) in any of the above analyses.

### Analyses of Snake Home Ranges

**Figure 2:**
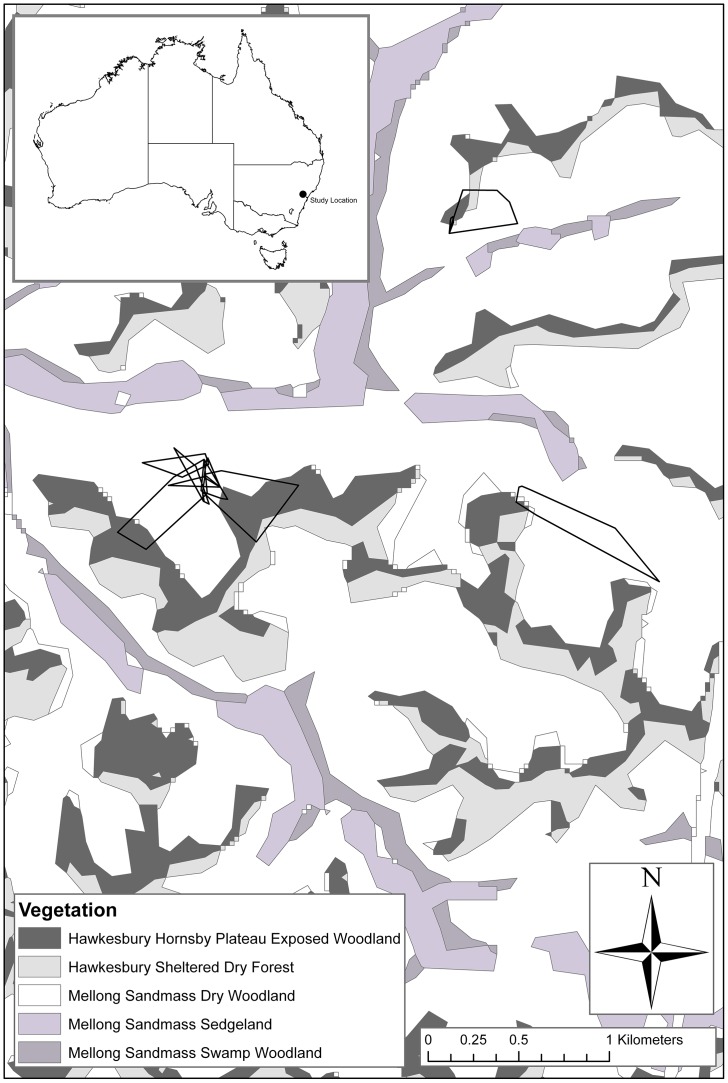
Home ranges of five adult female and three adult male broad-headed snakes that we radio-tracked. The home range boundaries were estimated using the minimum convex polygon method, with vegetation layer overlaid. All snakes that were tracked remained within “Mellong sandmass dry woodland” and “Hawkesbury–Hornsby plateau exposed woodland” and avoided all other vegetation types.

The mean home range of snakes throughout this study was 2.97±1.14 ha. Home range sizes differed between seasons: mean summer home range was 4.42±1.54 ha, and mean winter home range was 0.50±0.22 ha (see Table 1 for home range size for individual snakes and Fig. 2 for home range plots), although this difference was not significant due to low winter sample size.

### Analyses of Macrohabitat Use by Snakes

**Figure 3:**
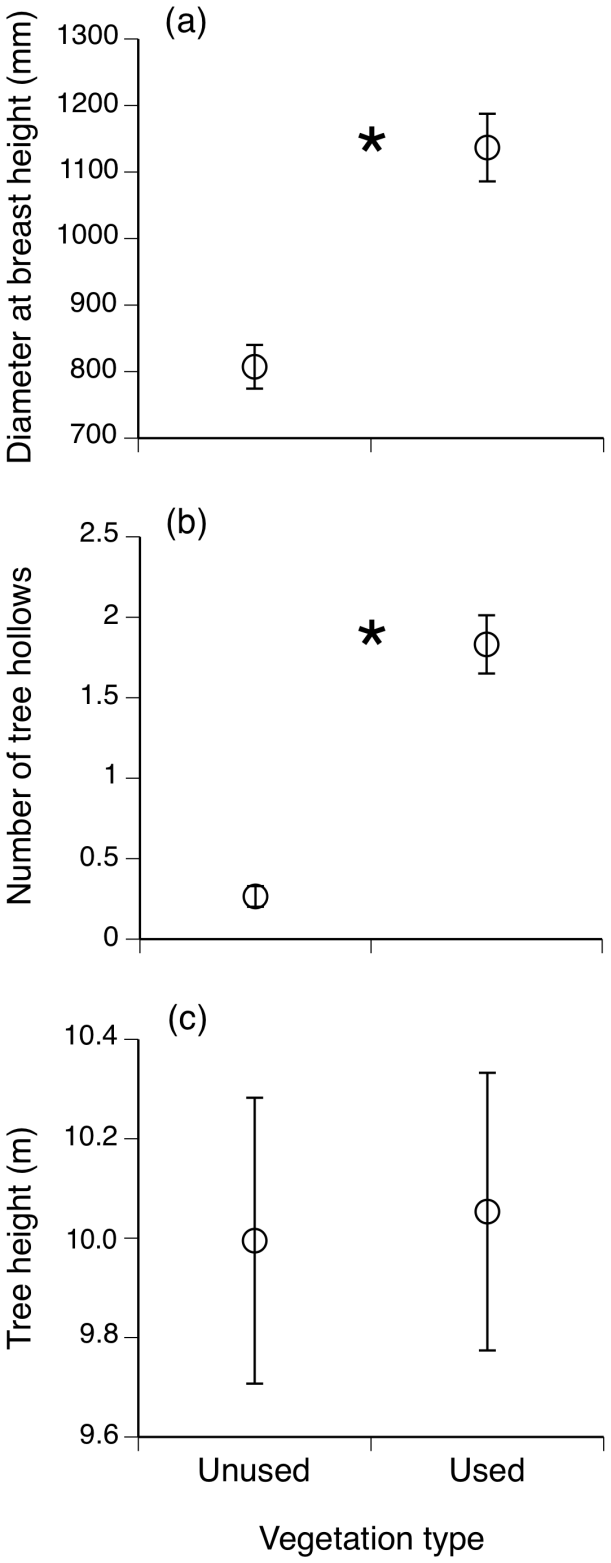
Characteristics of trees in vegetation types that were either used by radio-tracked broad-headed snakes (“Mellong sandmass dry woodland” and “Hawkesbury–Hornsby plateau exposed woodland”) or were not used by our snakes (“Hawkesbury sheltered dry forest”). In the preferred macrohabitat types, tree diameter at breast height (a) was greater and trees had more hollows (b). Mean tree height (c) did not differ between used and unused vegetation types. Graphs show mean values and associated standard errors. * indicates a statistically significant difference.

During summer, our radio-tracked snakes remained within two specific macrohabitat types: the “Hawkesbury–Hornsby plateau exposed woodland” and “Mellong sandmass dry woodland” (Fig. 2) where they used hollows in a variety of tree species, most notably red bloodwood, yellow bloodwood, grey gum, scribbly gum, narrow-leaved stringy bark and stags (standing dead trees). The snakes avoided the adjacent “Hawkesbury sheltered forest” (Fig. 2) which contained thinner trees (based on DBH, *t* = 5.44, df = 270, *P*<0.001; Fig. 3b) with fewer hollows per tree (*t* = 8.16, df = 218, *P*<0.001; Fig. 3a), but which were similar in mean height (*t* = 0.146, df = 247, *P* = 0.884; Fig. 3c) to those in the preferred habitat types.

### Analyses of Microhabitat Use by Snakes

A comparison between used and adjacent unused trees (all within the same habitat type) showed that snakes sheltered within a non-random subset of trees with respect to several variables. Our analysis of all combinations of variables produced 65 models, with 7 in the 95% confidence set (Σ*w_i_* = 0.95). Only 4 models had a ΔAICc <4. Compared to availability, snakes selectively used dead trees that were wider at the diameter at breast height, shorter and had many hollows relatively close to ground level (Table 2). The species of tree appeared to be less important than these structural features, with tree species not appearing in any of the highly-ranked models.

**Table 2:** Coefficients of the four best generalized mixed models and standard errors, with AICc values, change in AICc values (ΔAICc) and Akaike weight (*w_i_*).

Intercept	Alive or Dead	DBH	Hollow Height	# Hollows	Tree Height	AICc	ΔAICc	*w_i_*
−4.114	+	0.002±0.001	–	0.415±0.145	−0.198±0.102	94.499	0	0.425
−4.223	+	0.002±0.001	−0.086±0.124	0.451±0.154	−0.185±0.104	96.169	1.670	0.184
−5.425	+	0.001±0.001	–	0.327±0.134	–	96.672	2.173	0.143
−5.495	+	0.002±0.001	−0.134±0.123	0.394±0.148	–	96.934	2.435	0.126

**Figure 4:**
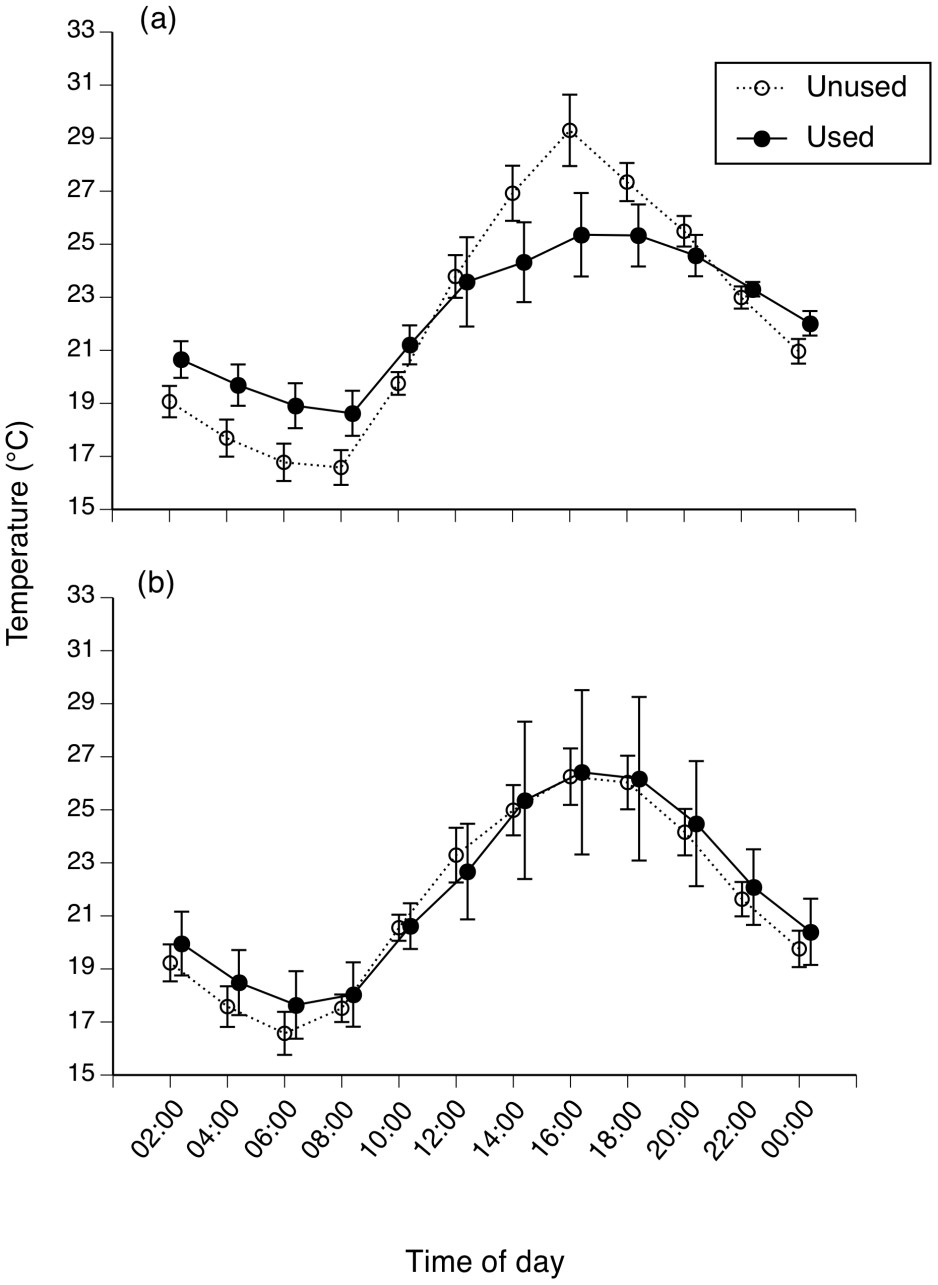
Thermal regimes in retreat-sites and potentially available retreat-sites. Panel (a) shows the temperature cycle on a hot day in the summer of 2010/2011 and (b) shows the same cycle on a hot day in the (cooler) summer of 2011/2012. Graphs show mean values and associated standard errors.

We found no significant difference in mean temperatures between used versus unused tree hollows (*F*_1,36_ = 0.10, *P* = 0.75), and no significant thermal difference between years (*F*_1,36_ = 0.65, *P* = 0.43; interaction NS also). Temperatures within a tree hollow shifted with time of day (*F*_11,26_ = 19.31, *P*<0.001; Fig. 4), with a significant interaction between time of day and year (*F*_11,26_ = 7.63, *P*<0.001: the summer of 2011–2012 was cooler than that of 2010–2011; Fig. 4). Maximum temperatures were higher in unused tree hollows than in hollows of used trees (*t* = 2.25, df = 15.9, *P* = 0.02).

### Seasonal Shifts in Thermal Regimes within Retreat Sites

**Figure 5:**
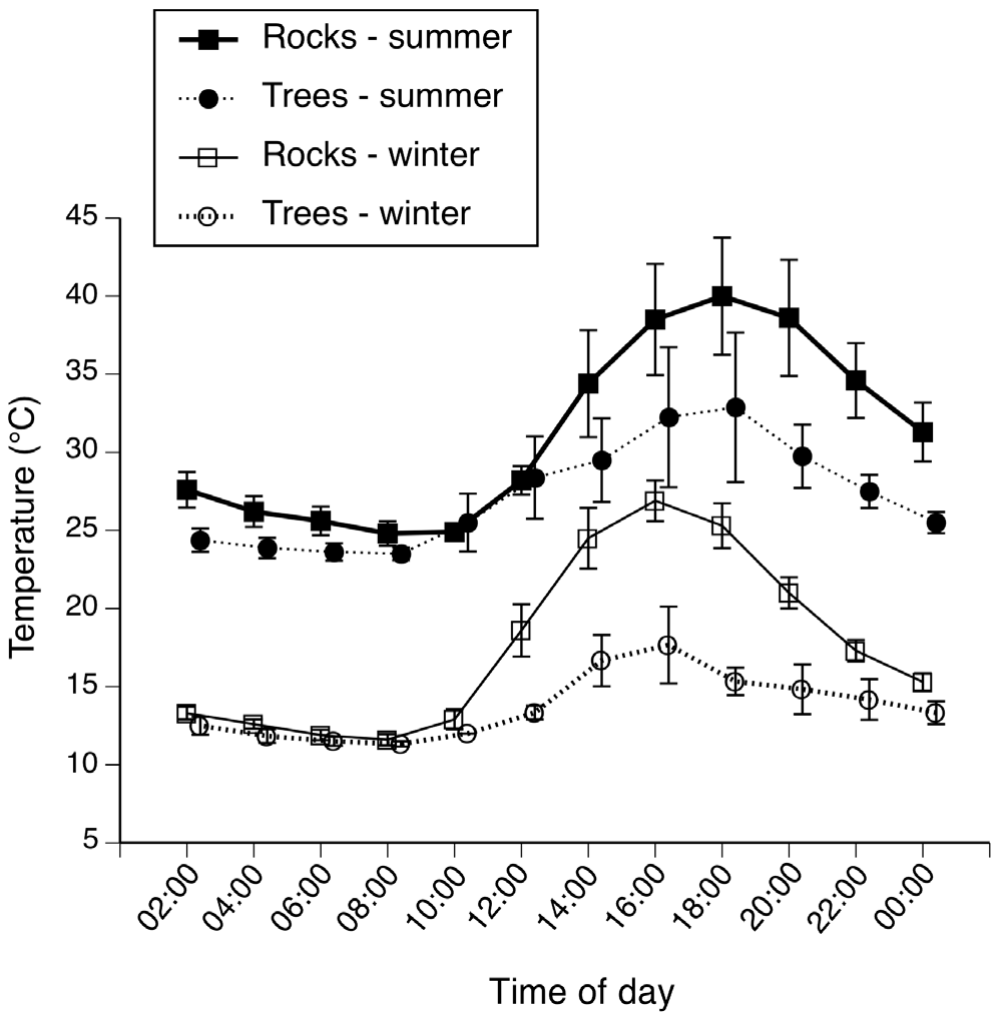
Mean maximum ambient temperatures experienced under rocks (winter habitat for broad-headed snakes) and in tree hollows (summer habitat for broad-headed snakes) during winter and summer. By shifting habitat between winter and summer, these snakes experience relatively stable thermal regimes and avoid extreme temperatures.

**Figure 6:**
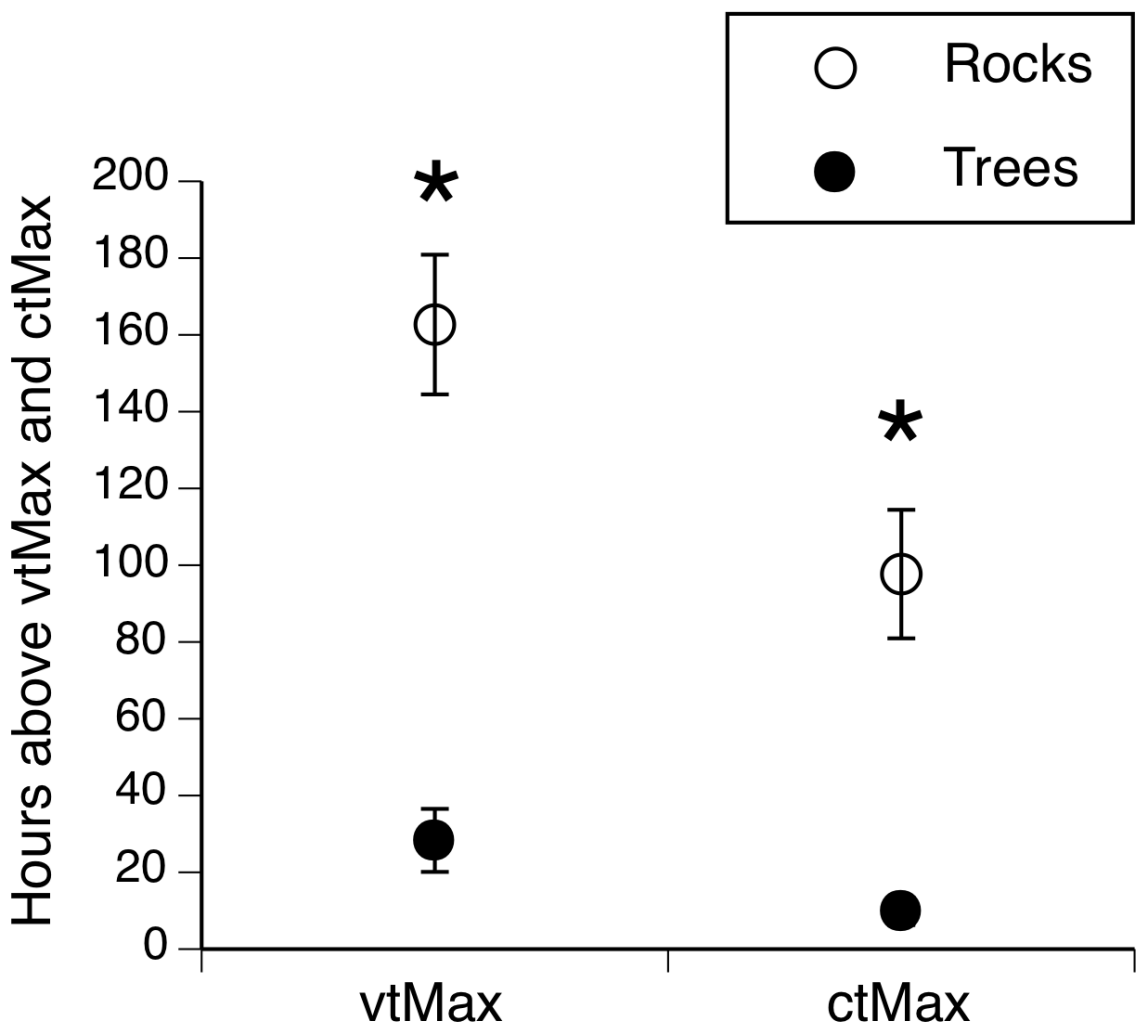
Thermal extremes in snake retreat-sites during the summer (1 December to 28 February) of 2010/2011. The graph shows the total number of hours at which retreat-site temperatures were above the voluntary thermal maximum (VTMax, 32.5°C) and estimated critical thermal maximum (CTMax, 38.0°C) of broad-headed snakes. Data are shown separately for two habitat types used by those snakes: those used in summer (tree hollows) and in winter (under rocks). Rocks often exceeded both VTMax and CTMax in summer, whereas tree hollows exceeded these limits only rarely. * indicates a statistically significant difference.

Mean temperatures under rocks differed from those inside tree hollows (*F*_1,13_ = 22.01, *P*<0.001; Fig. 5) and were higher in summer than in winter (*F*_1,13_ = 442.40, *P*<0.001; Fig. 5). Temperatures also differed with time of day (*F*_1,13_ = 106.932, *P* = 0.001; Fig. 5), but with no significant interaction between time of day and habitat type or time of day and season (all *P*>0.05). The number of hours during summer that CTMax (*F*_1,33_ = 20.12, *P*<0.001) and VTMax (*F*_1,33_ = 32.19, *P*<0.001) were exceeded was higher under rocks than in tree hollows, with rocks often exceeding those thermal limits (Fig. 6).

### Spatial Ecology in the North *versus* the South of the Species’ Range

Home range size did not differ significantly between northern and southern populations of *H. bungaroides* (*F*_1,33_ = 0.035, *P* = 0.852), nor did mean distance per move (*F*_1,33_ = 0.813, *P* = 0.375), nor total distances moved (*F*_1,33_ = 0.518, *P* = 0.476).

## Discussion

Our data reveal many similarities between *H. bungaroides* in southern populations (as previously studied) and those in the north of the range (the subject of our current work). For example, our radio-tracked snakes showed the same seasonal shift in habitat types, from rock crevices in winter to tree hollows in summer. Our data on thermal regimes within those habitat types support the hypothesis that these seasonal shifts are driven by temperature [17]. By moving between these habitat types seasonally, the snakes had access to relatively consistent thermal conditions inside refuges year-round (Fig. 5). Trees (summer habitat) are too cool to allow snakes to thermoregulate in winter, whereas sun-exposed rock crevices (winter habitat) attain lethally high temperatures in summer (see Figs 5 and 6). During the entire summer of 2010/2011, ambient temperatures in crevices (both under randomly-chosen rocks, and those actually used by *H. bungaroides* as winter retreat sites) exceeded the broad-headed snake VTMax for an average of 162.6 hours, and exceeded the snakes’ estimated CTMax for an average of 97.6 hours. In contrast, the temperatures that we measured inside randomly-chosen and used tree hollows exceeded VTMax for an average of only 28.3 hours, and exceeded CTMax for 9.8 hours (Fig. 6). Trees that were used by snakes never exceeded CTMax, and exceeded VTMax for only 9 hours throughout the summer. The hollows used by snakes were cooler then nearby unused hollows during the hottest parts of the day (see Fig. 4). We observed snakes basking outside tree hollows, and signal directions suggested that snakes moved up and down hollow limbs and trunks depending on ambient temperature. That mobility suggested active temperature selection by snakes within tree-hollows, similar to southern populations of the same species [21] and the more northerly-distributed congener, Stephen’s banded snake (*H. stephensii*: [37]). The smaller size of rocks precludes this kind of active behavioral thermoregulation, so the snakes’ shift to tree hollows in the warmer months of the year may reflect the advantages of greater accessible spatial thermal heterogeneity, as well as lower mean temperatures [21].

In terms of spatial ecology, the snakes we tracked in the extreme north of the species’ range were similar to conspecifics in southern populations. As well as the thermally-driven shifts in habitat noted above, mean home range sizes were similar for snakes in both regions (3.3 ha versus 3.0 ha: [17]), as were mean movement distances between successive displacements (134 m versus 159 m: [17]), the active selection of trees with many hollows and relatively large DBH [21], and fidelity for specific trees [21]. Like the southern snakes (J. K. Webb, unpublished data), our radio-tracked northern snakes sometimes were encountered under bushes and in hollow logs on the forest floor.

However, our data also identify some points of difference between snakes from the northern versus southern clades. In summer, southern conspecifics used trees on top of plateaus and below cliffs [17] whereas snakes in our northern study areas moved into shallow valleys away from rock outcrops, and occupied specific vegetation types while avoiding others. No such selectivity at this macrohabitat level has been recorded for the southern population, perhaps reflecting more homogeneous forest-habitat types in that region. The northern snakes showed several points of similarity with *H. stephensii*, an arboreal congeneric species that occurs north of the range occupied by *H. bungaroides* [27], [37]. Our study sites were close to the southern limit of *H. stephensii* distribution, and thus experience climatic conditions more similar to those encountered by southern *H. stephensii* populations than southern *H. bungaroides* populations. Thermal regimes may drive similarities such as occasional rock use by *H. stephensii* [38], as well as similar periods of sequestration inside retreat sites (mean of 7.9 days for *H. bungaroides* versus 8 days for *H. stephensii*: [39]). The southern *H. bungaroides* studied by Webb and Shine [17] moved much more frequently (mean of 2.9 days: [17]), suggesting that higher ambient temperatures may restrict the frequency of movement. Some specific trees were used by different individual snakes (both simultaneously and at different times) in our study, and snakes also used leaf litter as retreat sites. Both of these patterns were noted for *H. stephensii* [40], but rarely for southern *H. bungaroides* [17], [21]. In combination, these trends suggest that suitable arboreal shelter-sites may be more limiting in the north, such that in hot weather snakes may re-use a limited set of tree hollows, or else abandon arboreal sites for the cooler leaf litter.

The snakes that we radio-tracked used different species of trees for shelter than did the previously-studied southern population of *H. bungaroides* or *H. stephensii*, but this difference is most parsimoniously attributed to geographic differences in forest composition. In support of that inference, tree species seemed to have less effect than tree structure in determining frequency of use by snakes. The four generalized mixed models with best fit to our data (Table 2) suggested that snakes prefer trees that were dead, with a large DBH but were also fairly short with many hollows close to the ground. In contrast, the tallest available trees were preferred by both southern-clade *H. bungaroides* and more northern *H. stephensii* [21], [40]. That difference in tree height may explain an otherwise-puzzling discrepancy between our study and the earlier work: that is, we saw snakes basking in exposed positions on trees a total of 9 times (including 5 different snakes), whereas this behavior was rarely observed in the studies on other *Hoplocephalus* [21], [40]. Overt basking may be more likely on shorter trees (because they tend to be mostly in shade due to adjacent taller trees); and also, basking may be easier to observe if the snakes are closer to the ground (and thus, to the observer).

During our study, three trees were used by more than one snake, with up to four individual snakes using the same tree throughout both summers and the winter tracking period. Two of these trees were inhabited by two snakes simultaneously, a phenomenon also observed (albeit rarely) in *H. stephensii* [40] but not in southern clade *H. bungaroides*. Northern clade *H. bungaroides* showed strong site fidelity, as also reported in southern clade *H. bungaroides* and *H. stephensii*. We observed five snakes return to the same trees, with one snake using the same tree on four occasions throughout the summer of 2010/2011. Such re-use suggests that trees with suitable hollows a limiting resource for snakes in this system.

Broadly, the habitats used by our northern-clade *H. bungaroides* were similar to those used by southern-clade conspecifics in winter. The reliance on hollow-bearing trees during summer is shared not only by the two *H. bungaroides* clades, but also by two congeneric arboreal taxa (*H. stephensii* and *H. bitorquatus*, the pale-headed snake: [39], [40]). Although detailed studies are lacking, arboreality and tree-hollow use also are likely to be important for the most closely related outgroup taxa to *Hoplocephalus* – *Tropidechis carinatus* (the rough-scaled snake) and *Paroplocephalus atriceps* (the Lake Cronin snake: [36], [41], [42]. Phylogenetic reconstructions suggest that *H. bitorquatus* most closely resembles the ancestral *Hoplocephalus* species [41]; hence, year-round arboreality may be an ancestral trait for this lineage. The expansion of the southernmost taxon (*H. bungaroides*) into cooler areas rendered local tree-hollows too cool for foraging in winter, plausibly stimulating a behavioral shift towards rock-crevice use during cooler times of year.

Our results have direct implications for conservation and management of the genetically distinct northern clade of *H. bungaroides*. First, the population that we studied relies upon both rock outcrops (in winter) and nearby forests (in summer), so management needs to conserve that combination of habitat types in close proximity. That requirement is similar to that for southern conspecifics, whereas rock crevices appear to play only a minor role in the ecology of the other *Hoplocephalus* species [40]. In terms of conserving rock-outcrop habitats, attention needs to focus not only on human disturbance to local areas (especially, rock theft for landscaping, and illegal collection of animals for the pet trade: [15], [16]) but also on broader landscape-scale processes. Analyses of historical photographs, and long-term field studies, have shown that vegetation overgrowth imperils *H. bungaroides* at the southern study sites [20]. Removal of shading vegetation significantly enhanced habitat quality for *H. bungaroides* in this area [19]. We have no equivalent data for the northern-clade populations, but they may well be under similar threats (e.g., illegal rock collection is rife: B. Croak, unpublished data). Thus, management should prioritize retention of existing surface rock, and mitigation of processes that facilitate vegetation overgrowth [14], [15], [19], [20], [22].

Because *H. bungaroides* show such a profound seasonal shift in habitat use, we also need to maintain large forest blocks that contain hollow-bearing trees, in areas adjacent to sandstone outcrops [21], [37]. For the northern population, that forested area should lie within vegetation types such as “Hawkesbury–Hornsby plateau exposed woodland” and “Mellong sandmass dry woodland”, rather than other locally occurring forest types. The preferred macrohabitats may be distinctive because they contain a relatively high number of trees with large hollows suitable for retreat-site use by *H. bungaroides*. The same types of hollows are used by many other arboreal taxa, emphasizing the importance of this critical habitat for a wide variety of species [43], [44]. Thus, forestry management plans should aim to conserve tree hollows. In Australia, managed landscapes generally support less than half the number of hollow-bearing trees as occur in natural stands [43]. This issue may be especially critical for the northern populations of *H. bungaroides*, because their frequent re-use of the same tree hollows, and use of those hollows by multiple animals, suggests that such trees may be a limiting resource in this system.

The spatial extent of reserves to protect northern-clade *H. bungaroides* is likely to be similar to that needed for their southern-clade conspecifics. Movement patterns of northern *H. bungaroides* were broadly similar to those of their southern conspecifics. Rates of gene flow also are likely to be similar in the two clades [45], with a complex metapopulation structure that includes unidirectional gene flow from source to peripheral sink populations [45]. Identification of source and sink populations through genetic investigation within the northern range of this endangered species would facilitate effective conservation and/or habitat restoration [45]–[47].
